# Cryo-EM structure of Nipah virus RNA polymerase complex

**DOI:** 10.1126/sciadv.adr7116

**Published:** 2024-12-11

**Authors:** Yiru Wang, Lixia Zhao, Yi Zhang, Yuhan Wang, Jiao Tang, Simiao Liu, Huihan Gao, Xiaoxiao Zhang, Luca Zinzula, Roger D. Kornberg, Heqiao Zhang

**Affiliations:** ^1^ Shanghai Institute for Advanced Immunochemical Studies, ShanghaiTech University, 201210, Shanghai, China.; ^2^ School of Life Science and Technology, ShanghaiTech University, 201210, Shanghai, China.; ^3^ iHuman Institute, ShanghaiTech University, 201210, Shanghai, China.; ^4^ State Key Laboratory of Resource Insects, Institute of Apicultural Research, Chinese Academy of Agricultural Sciences, 100193, Beijing, China.; ^5^ State Key Laboratory of Plant Genomics, Institute of Genetics and Developmental Biology, Chinese Academy of Sciences, 100101, Beijing, China.; ^6^ Department of Structural Biology, Stanford University School of Medicine, Stanford, CA 94305, USA.

## Abstract

Nipah virus, a member of the *Paramyxoviridae* family, is a highly pathogenic nonsegmented, negative-sense RNA virus (nsNSV) which causes severe neurological and respiratory illnesses in humans. There are no available drugs or vaccines to combat this virus. A complex of large polymerase protein (L) and phosphoprotein (P) of Nipah virus supports replication and transcription and affords a target for antiviral drug development. Structural information required for drug development is lacking. Here we report the 2.9-angstrom cryo–electron microscopy structure of the Nipah virus polymerase-phosphoprotein complex. The structure identifies conserved amino acids likely important for recognition of template RNA by nsNSVs and reveals the locations of mutation-prone sites among Nipah virus strains, which may facilitate the development of therapeutic agents against Nipah virus by targeting regions unaffected by these mutation sites.

## Teaser

The structure of the Nipah virus polymerase complex reveals key structural features essential for viral transcription.

## INTRODUCTION

Nipah virus (NiV), a zoonotic nonsegmented, negative-sense RNA virus (nsNSV), belongs to the *Paramyxoviridae* family of the order *Mononegavirales* (*1*). It was first identified in Malaysia and Singapore in 1998–1999 (*2*) and has caused several outbreaks in South or Southeast Asia (*3*–*6*). In Malaysia, there were 265 reported cases of encephalitis, with a fatality rate of approximately 40% (*7*).

NiV, whose wildlife reservoir is *Pteropus* bats (*8*), is classified in the genus *Henipavirus*, which also includes the Hendra virus (HeV) (*7*, *9*). Both NiV and HeV induce severe neurological and/or respiratory diseases in humans (*10*). Since its emergence in Malaysia, NiV spread efficiently in Bangladesh, India, and, most recently, in China, through frequent person-to-person transmission (*3*–*6*, *11*, *12*), elevating the mortality rate to more than 70% (*13*, *14*). Previous clinical studies demonstrated that approximately one-third of NiV-infected survivors suffer permanent neurological deficits (*15*). NiV exhibits a broader host range than other members of the *Paramyxoviridae* family (*16*, *17*), thus posing a considerable risk of a global pandemic. No effective antivirals or vaccines are available to treat NiV infection.

The genome of NiV encodes six proteins: fusion protein (F), glycoprotein (G), matrix protein (M), nucleocapsid (N), phosphoprotein (P), and large polymerase protein (L) (*18*, *19*). The complex formed by the L and P proteins serves as both replication and transcription machineries. According to sequence analysis, the Nipah L protein harbors five distinct domains, an N-terminal domain (NTD), an RNA-dependent RNA polymerase (RdRp) domain, a guanosine diphosphate polyribonucleotidyltransferase (PRNTase) domain, a methyltransferase (MTase) domain, and a C-terminal domain (CTD). The P protein forms tetramers that bridge between the L and N proteins in NiV as well as in other *Paramyxoviridae* family members (*20*). NiV P is much larger than the P protein of other *Paramyxoviridae* family members. The RdRp domain of the L protein, responsible for the synthesis of both viral genomic RNA and mRNA (*21*, *22*), represents an attractive target for antiviral drug development.

High-resolution structures of the L-P complexes of other nsNSVs, such as the Ebola virus (EBOV) (*23*, *24*), respiratory syncytial virus (RSV) (*25*–*27*), vesicular stomatitis virus (VSV) (*28*, *29*), human metapneumovirus (HMPV) (*30*), and parainfluenza virus 5 (PIV5) (*31*), have been reported. Structural information on the NiV L-P complex, however, has remained elusive, impeding progress in antiviral drug development. Here, we report the cryo–electron microscopy (cryo-EM) structure of NiV L-P complex at a resolution of 2.9 Å.

## RESULTS

### Overall structure of the NiV L-P complex

**Fig. 1.**
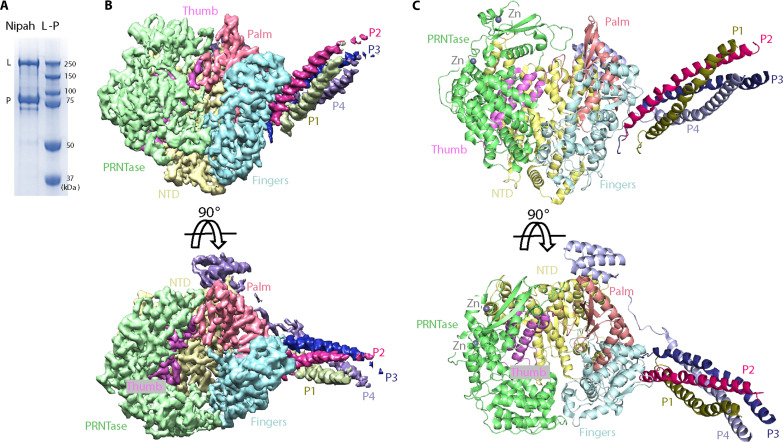
Purification and cryo-EM analysis of the NiV L-P complex. (**A**) SDS–polyacrylamide gel electrophoresis analysis of the purified NiV L-P complex. The sample after size exclusion chromatography was loaded on a 10% resolving gel and stained with Coomassie blue. (**B**) Segmentation of the NipV L-P reconstruction map, rotated by 90°. The NTD, fingers, palm, thumb, and PRNTase domains of Nipah-L are colored in yellow, cyan, salmon, violet, and green, respectively. The four protomers of Nipah-P, P1, P2, P3, and P4, are depicted in deep olive, firebrick, deep blue, and light blue, respectively. (**C**) An atomic model of the NiV L-P complex. Different domains of Nipah-L and four protomers of Nipah-P are color-coded according to the segmentation maps, rotated by 90°. Two zinc ions are shown as gray spheres.

**Fig. 2.**
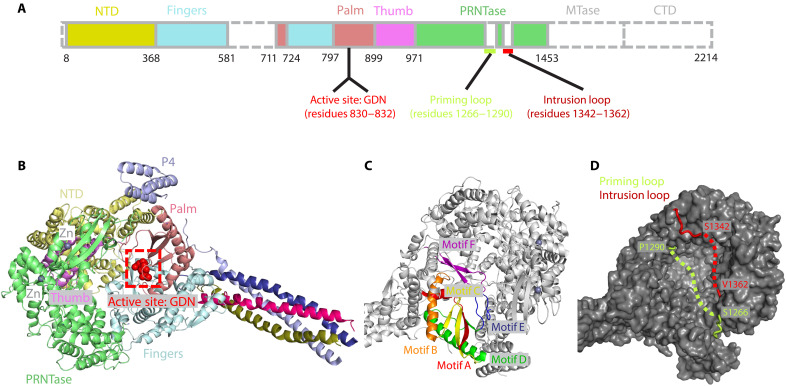
Detailed structural analysis of the active center of the Nipah-L protein. (**A**) Schematic diagram of the domain organization of Nipah-L. Residues at domain boundaries are labeled. The active site amino acids, priming loop, and intrusion loop are specified at the bottom of the scheme. (**B** and **C**) Detailed structure of the active center. The GDN motif is shown as red spheres, and the motifs A to F are colored in red, orange, yellow, green, blue, and purple, respectively. (**D**) Disordered priming and intrusion loops in the Nipah L structure. The priming and intrusion loops are shown as ribbon diagram and colored in limon and red, respectively. The boundary residues, including Ser^1266^, Pro^1290^, Ser^1342^, and Val^1362^, are indicated, and the disordered regions are indicated with dashed lines.

Recombinant full-length NiV L-P complex was overexpressed in insect cells and purified to homogeneity by anti-FLAG affinity, heparin affinity, and size exclusion chromatography (Fig. 1A and fig. S1A). The purified NiV L-P complex retains RdRp enzymatic activity for de novo RNA synthesis in vitro, as demonstrated by its capability to hydrolyze nucleoside triphosphate (NTP) moieties into monophosphate ones in the presence of a synthetic single-stranded RNA (ssRNA) template, which was monitored by a fluorescence-based pyrophosphate assay (fig. S1B). High-quality cryo-EM data were collected on a Titan Krios electron microscope equipped with a Falcon 4 electron detector (fig. S1, C and D). Data processing performed in cryoSPARC (*32*) resulted in a 2.9-Å reconstruction map (Fig. 1B and fig. S2), leading to an atomic model for the NiV L-P complex (Fig. 1C, fig. S3, and table. S1). As anticipated, an L protein with a P protein tetramer was observed, with varying lengths of the P proteins visible in the cryo-EM map (Fig. 1C). The NTD, RdRp, and PRNTase domains of the L protein were visible (Fig. 1C), but the MTase domain and the CTD were not, likely due to motion. The RdRp domain of NiV L adopts a characteristic right-hand “fingers-palm-thumb” fold, with the “thumb” subdomain sandwiched by the NTD and PRNTase domains (Fig. 1C). Two zinc ions, coordinated by a “Cys-Cys-Cys-Glu” motif and a “Cys-Cys-His-His” motif, compact the PRNTase domain (Fig. 1C), reminiscent of the features observed in PIV5 (*31*) and VSV L-P complexes (*28*). In addition, a “G-D-N” motif (residues 830 to 832), supposed to be the active site of the L protein (*33*), is situated in the loop region between β strand 1 and β strand 2 of the “palm” domain (Fig. 2, A and B). Therefore, our structure reveals all crucial motifs identified in L proteins from other nsNSVs, that also in NiV are likely crucial for NTP binding and catalysis, extending from “motif A” to “motif F” (Fig. 2C). Unlike the “retracted” and “engaged” conformations reported in other nsNSV structures, the priming loop, known to be essential for RNA synthesis (*23*, *34*, *35*), and the intrusion loop, required for PRNTase activity among nsNSVs (*36*), are not visible in our cryo-EM map (Fig. 2D), reminiscent of the disordered state of both loops observed in the EBOV L-VP35 structure when RNA is not present (*23*). The different conformations of the priming and intrusion loops observed in nsNSV members indicate various standby states used by nsNSVs to initiate RNA synthesis and capping.

### Interaction between polymerase and phosphoproteins

**Fig. 3.**
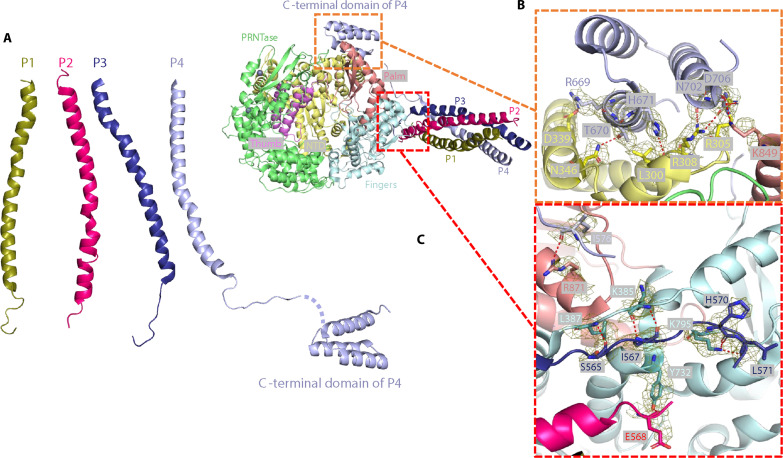
Detailed interactions between the Nipah-L and the four protomers of Nipah-P. (**A**) Structures of the four Nipah-P protomers. (**B** and **C**) Close-up views of the interactions between Nipah-L and Nipah-P protomers. Interacting residues are shown in stick representation. The electron density maps of the interacting residues are shown in mesh (contour level 4σ). Single-letter abbreviations for the amino acids shown in the figure are listed as follows: A, Ala; D, Asp; E, Glu; F, Phe; H, His; I, Ile; K, Lys; L, Leu; N, Asn; S, Ser; T, Thr; R, Arg; Y, Tyr.

Similar to previously available structures of L-P complexes from nsNSVs, such as PIV5 (*31*), EBOV (*23*), RSV (*25*, *26*), and VSV (*29*), the four proteins of the NiV P tetramer, here designated as “P1,” “P2,” “P3,” and “P4,” form a left-hand helical bundle, contacting the “fingers” subdomain of the L protein (Fig. 3A). One interface between the L protein and the tetrameric P proteins is located at the base of the helical bundle; the other interface is formed by the NTD domain of the L protein and the CTD of P4 (Fig. 3, B and C). The interface at the base of the helical bundle involves a hydrogen bond network formed by P proteins P2, P3, and P4 with the fingers subdomain of the L protein. Specifically, the main chain of Ile^576^ in P4 forms a hydrogen bond with Arg^871^ in the palm subdomain (Fig. 3C). A C-terminal extension of P3 interacts extensively with the fingers subdomain of L, engaging residues Ser^565^, Ile^567^, His^570^, and Leu^571^ of P3 and Lys^385^, Leu^387^, and Lys^795^ of L (Fig. 3C). A hydrogen bond formed by the main chain of Glu^568^ in P2 and Tyr^732^ within the fingers subdomain further strengthens the L-P interaction (Fig. 3C). The CTD of P4, consisting of three α helices, interacts extensively with the NTD domain of the L protein, primarily through hydrogen bonds and salt bridges (Fig. 3B).

### Entry and exit channels for template RNA, nascent RNA, and incoming NTP

**Fig. 4.**
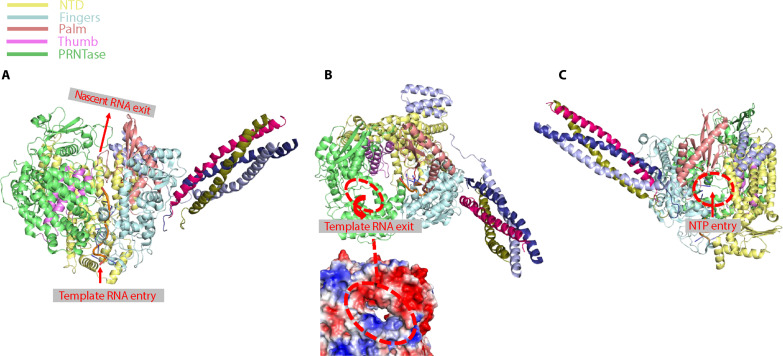
Entrances and exits for template RNA, nascent RNA, and NTPs. (**A**) Entrance for template RNA and exit for nascent RNA, highlighted by red arrows. (**B**) Top: Exit channel for template RNA on Nipah-L, indicated by a red dashed circle and an arrow. Bottom: Close-up view of the exit channel, shown as the electrostatic surface. (**C**) Entry channel for incoming NTPs on Nipah-L, indicated by a red dashed circle. Different domains of Nipah-L and the protomers of Nipah-P are color-coded.

The structure of the NiV L-P complex resembles a funnel. Superposition of our structure with that of template RNA-bound L-P complex from RSV identifies an entry channel for template RNA in the NiV L protein at the bottom of the funnel (Fig. 4A). Previous studies have indicated that the exit channel for template RNA resides in the PRNTase domain (*24*, *27*). We have identified a charged channel on the PRNTase domain that may act as the exit route for template RNA (Fig. 4B). Aligning our structure with that of a rotavirus L-P complex reveals a path for exit of nascent RNA (Fig. 4A). The 5′ end of nascent RNA likely extends from this exit channel to reach the MTase domain, not visible in our structure, for further modification. An NTP entry channel is created by the “NTD,” palm, and fingers subdomains of the NiV L protein, with the three C-terminal α helices of NiV P4 adjacent to the entrance for NTP (Fig. 4C).

### Conserved amino acids in nsNSVs required for template RNA recognition

**Fig. 5.**
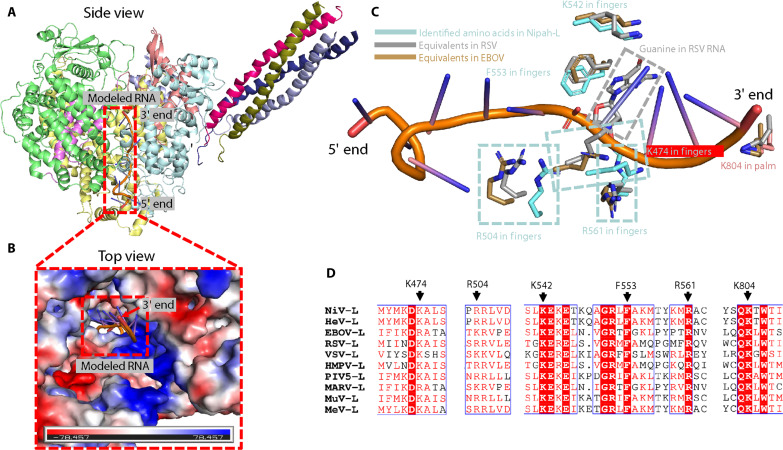
Predicted model for template RNA-bound NiV L-P complex. (**A**) Side view of the predicted model of template RNA-bound L-P complex, with the template RNA highlighted in a red dashed box. The template RNA from the RSV L-P structure was rigidly docked into the Nipah-L cavity by aligning the two structures. For simplicity, only the RNA bound in the RSV L-P complex is shown. (**B**) Top view of the predicted model. The modeled RNA is highlighted in a red dashed box, and the NiV L-P complex is shown as the electrostatic surface. (**C**) Structural superposition of the identified conserved amino acids essential for template RNA binding with those of EBOV and RSV. The amino acids are shown in stick representation, with the amino acids from the fingers subdomain and Lys^804^ from the palm subdomain of Nipah-L colored in cyan and salmon, respectively. The equivalents in RSV and EBOV are color-coded. (**D**) Sequence alignment of the identified amino acids with those of HeV, EBOV, RSV, VSV, HMPV, PIV5, MARV, MuV, and MeV viruses. The amino acids are indicated by black arrows.

The only nsNSV structures of template RNA-bound L-P complexes come from EBOV (*24*) and RSV (*27*). When the EBOV and RSV structures were aligned with the one reported here, an essentially perfect fit of the template RNA in the NiV template entry channel was observed (Fig. 5, A and B; for simplicity, only the RNA bound in the RSV L-P complex is shown). Six amino acids that line the channel, Lys^474^, Arg^504^, Arg^542^, Phe^553^, Arg^561^, and Lys^804^, are conserved among the EBOV, RSV, and NiV structures (Fig. 5, C and D). The five positively charged amino acids likely interact with the RNA backbone, while the sixth, Phe^553^, interacts with RNA bases (guanine in EBOV and cytosine in RSV RNA, respectively) through π-π interactions. Sequence alignment of the NiV L protein with those from other representative nsNSVs, such as HeV, EBOV, RSV, VSV, HMPV, PIV5, Marburg virus (MARV), Mumps virus (MuV), and Measles virus (MeV), shows that these amino acids are highly conserved. Notably, Arg^542^, Phe^553^, Arg^561^, and Lys^804^ in the NiV protein, along with their equivalents in these aforementioned viruses, are strictly conserved (Fig. 5D). These six highly conserved amino acids that we identified in this study may constitute the minimal set required for recognition of single-stranded template RNA among nsNSVs. In addition to the conserved amino acids that may be necessary for template RNA recognition, other conserved amino acids are mainly located in motifs A to F (fig. S4, A and B), which are in close proximity to the 3′ end of modeled RNA and active site amino acids “GDN” (fig. S4B), suggesting that these conserved amino acids are likely crucial for RNA synthesis.

### Sites of mutations in polymerase proteins among NiV strains

**Fig. 6.**
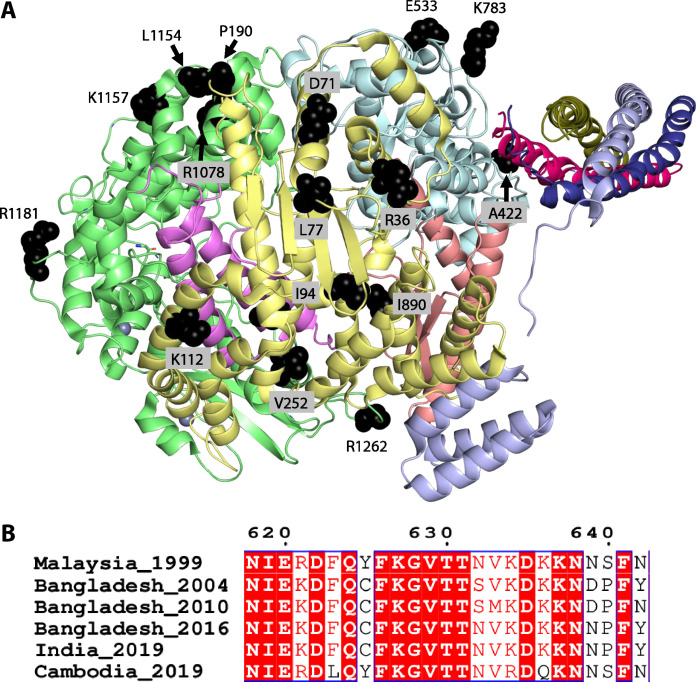
Mapping of mutation-prone sites across different Nipah strains. (**A**) Representation of mutation-prone sites on the Nipah-L protein. The amino acids are shown as spheres and colored black. (**B**) Sequence alignment for the most variable region of the Nipah-L protein.

Since the initial outbreak of NiV infection in Malaysia, outbreaks have occurred in various Asian countries, including Singapore, Bangladesh, India, Cambodia, and, most recently, China. Nipah genome, and especially its L protein, has accumulated multiple mutations during these outbreaks. We mapped the mutations in L proteins from NiV strains collected in Malaysia (1999), Bangladesh (2004, 2010, and 2016), India (2019), and Cambodia (2019) onto the Nipah L protein structure. The L proteins from these NiV strains exhibit high sequence identity (>90%), with the majority of mutations situated on the outer surface of the protein, distant from the catalytic cavity or the interfaces with P proteins (Fig. 6A). Moreover, the most variable region lies within the concealed loop (residues 621 to 642) between the fingers subdomain and motif A of the palm subdomain (Fig. 6B). We therefore provide a structural template for rational design of antiviral drugs against NiV by targeting regions unaffected by these mutations.

## DISCUSSION

Polymerase complex structures of nsNSVs have been reported for EBOV (*23*, *24*), RSV (*25*, *26*), VSV (*29*), HMPV (*30*), and PIV5 (*31*). Comparison of these structures with that of the NiV L-P complex reported here revealed a high degree of structural conservation among nsNSVs (fig. S5), suggesting conserved transcription and replication mechanisms shared by the nsNSVs. Furthermore, the conserved GDN motif crucial for coordinating two catalytic magnesium ions not only retains sequence similarity but also exhibits structural conservation, suggesting a common active center architecture where replication and transcription processes occur. It is important to note that while the L-P complexes from the nsNSV family share a similar overall architecture, determining the high-resolution structure of each member is crucial, particularly due to the need for precise structures in drug development.

As for L-P complex structures from other nsNSVs, the C-terminal region of the Nipah L protein, including the MTase and CTD domains, was not visible in the reconstruction map. AlphaFold2 was used to predict the structure of the PRNTase-MTase-CTD region of the NiV L protein, which was then aligned with the structure of the NTD-RdRP-PRNTase region reported here by alignment of the PRNTase regions. The overall architectures of the AlphaFold2-predicted PRNTase model and the PRNTase domain of our structure were similar, enabling the proposal of an entire L protein structure. The MTase and CTD domains in this structure are seen to enclose the open chamber formed by the NTD, RdRP, and PRNTase domains, serving as a “lid” on the structure (fig. S6A). All domains and subdomains of the L protein collaborate to form the entry and exit tunnels for template RNA and product RNA (fig. S6B), thereby initiating and regulating NiV transcription and replication. The MTase and CTD domains of the NiV L protein might be stabilized by the nascent RNA during replication or transcription. It is worth noting that our efforts to capture the NiV polymerase complex in an initiation or elongation state by incubating the purified proteins with the template RNA used for the pyrophosphate assay were unsuccessful. Future work is needed to address the stability issue of nsNSVs’ polymerase-RNA complexes.

## MATERIALS AND METHODS

### Overexpression and purification of the NiV L-P complex

The genes encoding the NiV L (GenBank: AAK29089.1) and P (GenBank: AAF73378.1) were codon-optimized and synthesized from GenScript Biotech. The synthesized NiV L and P open reading frames were individually cloned into the pFastBac1 vector, with the L subunit bearing a FLAG tag at its N terminus to facilitate affinity purification. Expression and purification of the NiV L-P complex were performed as previously described (*37*, *38*), with slight adjustments. Baculoviruses were generated using the standard Bac-to-Bac baculovirus expression system from Invitrogen. The NiV L-P complex was coexpressed in High Five cells (cultured in ESF921 medium at a density of 1.0 × 10^6^ cells ml^−1^) by infecting with equal volumes of L and P baculoviruses for 48 hours at 27°C. Following infection, 2 liters of infected High Five cells was harvested and centrifuged for 20 min at 4°C (1000 g), and the cell pellet was resuspended in a buffer containing 25 mM Hepes (pH 8.0), 500 mM NaCl, 4 mM MgCl_2_, and 3 mM dithiothreitol (DTT). The cells were then lysed by sonication, and the lysate was further cleared by 1-hour high-speed centrifugation (30,000*g*, 4°C). The cleared lysate was purified using anti-FLAG resins (GenScript Biotech), followed by purifications using heparin and size exclusion chromatography (Superose 6, Cytiva) in a buffer containing 25 mM Hepes (pH 8.0), 500 mM NaCl, and 3 mM DTT. The peak fractions containing the L-P complex were analyzed by SDS–polyacrylamide gel electrophoresis, and the best fractions were pooled and buffer-exchanged to 25 mM Hepes (pH 8.0), 100 mM NaCl, and 3 mM DTT. The purified proteins were concentrated to approximately 3 mg/ml stock solution to be used for the pyrophosphate assay and to 0.5 mg/ml for cryo-EM experiments, respectively. The proteins were aliquoted, flash-frozen, and stored at −80°C until further use.

### NiV L-P pyrophosphate assay

Purified NiV L-P complex was diluted to 0.5 mg/ml in a volume reaction of 50 μl [50 mM tris HCl (pH 7.5), 50 mM NaCl, 5 mM MgCl_2_, and 0.006% Tween 20] and incubated at 30°C for up to 1.5 hours in the presence of 100 μM NTP and a 1.0 μM 18-nt-long synthetic ssRNA (5′-UAUUCUCCCUUGUUUGGU-3′) template (GenScript Biotech). Pyrophosphate release resulting from NTP hydrolysis and putative nucleotide monophosphate (NMP) incorporation by NiV L-P into de novo ssRNA was monitored by fluorescence detection on a 384-well format, at 563-nm excitation and emission wavelength maxima, using the PiPer PyroPhosphatase assay kit (Thermo Fisher Scientific) according to the manufacturer’s instructions. Specificity of RdRp activity was assessed by subtracting fluorescence counts of the same reaction in the absence of the L-P complex, containing ssRNA and NTPs only, and expressing it as percentage of relative fluorescence compared to that of a standard reaction containing the same concentration of pyrophosphate as NTP moiety. Experimental data points were calculated as the mean and SDs of at least four independent replicates.

### Cryo-EM grid preparation

Grid preparations were performed as previously described (*37*, *38*) using a Vitrobot Mark IV. The 300-mesh Quantifoil R1.2/1.3 Au grids were glow-discharged for 30 s before grid plunge freezing. Three microliters of purified NiV L-P complex (0.5 mg/ml) was applied to the grid, then blotted for 2.5 s (blot force: −1), and subsequently plunged into liquid ethane with 100% chamber humidity at 8°C. All grids were stored in liquid nitrogen dewar until data collection.

### Cryo-EM data collection and image processing

Automated data acquisition was performed using EPU (Thermo Fisher Scientific) on Titan Krios equipped with a Selectris energy filter and a Falcon 4 direct electron detector (Thermo Fisher Scientific) operating at 300 kV, with a nominal magnification of ×130,000 and a pixel size of 0.96 Å (counting mode). A total of 5795 micrographs were automatically recorded in counting mode, with a defocus range from −1.3 to −2.3 μm. Each movie stack was dose-fractionated to 40 frames with a total electron dose of approximately 50 e^−^/Å^2^ and a total exposure time of approximately 6 s. Data processing was performed in cryoSPARC as previously described (*37*, *38*). Movie stacks were motion-corrected using Patch Motion Correction, and the defocus value was estimated using Patch CTF Estimation in cryoSPARC (*32*). Particle picking was initially performed using Blob Picker and filtered using Inspect Picks in cryoSPARC (*32*). The picked particles were then extracted with a box size of 256 pixels (bin = 2), followed by two rounds of two-dimensional (2D) classification, generating a template for subsequent Topaz Train–based picking. A total of 1,405,344 particles picked from Topaz Train were extracted with a box size of 256 pixels (bin = 2) and then subjected to two rounds of 2D classification. Subsequently, 523,611 particles selected from the best classes of the last round 2D classification were reextracted with a box size of 280 pixels (bin = 1) and subjected to ab initio reconstruction (number of classes = 4), followed by heterogeneous refinement. Homogeneous refinement and nonuniform refinement were sequentially performed for the best class selected from the heterogeneous refinement, resulting in a reconstruction map of NiV L-P complex at 2.9 Å.

### Model building and figure preparation

All the model building work was performed using the cryo-EM module of Phenix package (*39*), assisted by Chimera (*40*) and COOT (*41*). The AlphaFold2-predicted models of the separate domains of Nipah L and P proteins were docked into the cryo-EM map and manually adjusted in COOT (*41*), followed by several rounds of real-space refinement using Phenix (*39*). All the structure figures were generated using PyMOL (https://pymol.org/2/) and Chimera.
