# Supplementary material for: Cryo-EM structure of Nipah virus RNA polymerase complex
Source: Sci Adv. 2024 Dec 11;10(50):eadr7116. doi: 10.1126/sciadv.adr7116 (PMC11633731; doi:10.1126/sciadv.adr7116)
Supplement: Supplementary file 1 — Figs. S1 to S6 Table S1 [file sciadv.adr7116_sm.pdf]

Science Advances
AAAS

Supplementary Materials for

Cryo-EM structure of Nipah virus RNA polymerase complex

Yiru Wang *et al.*

Corresponding author: Heqiao Zhang, zhanghq@shanghaitech.edu.cn; Roger D. Kornberg, kornberg@stanford.edu; Luca Zinzula, zinzulal@shanghaitech.edu.cn; Xiaoxiao Zhang, zhangxx4@shanghaitech.edu.cn

Sci. Adv. **10**, eadr7116 (2024)
DOI: 10.1126/sciadv.adr7116

This PDF file includes:

Figs. S1 to S6
Table S1

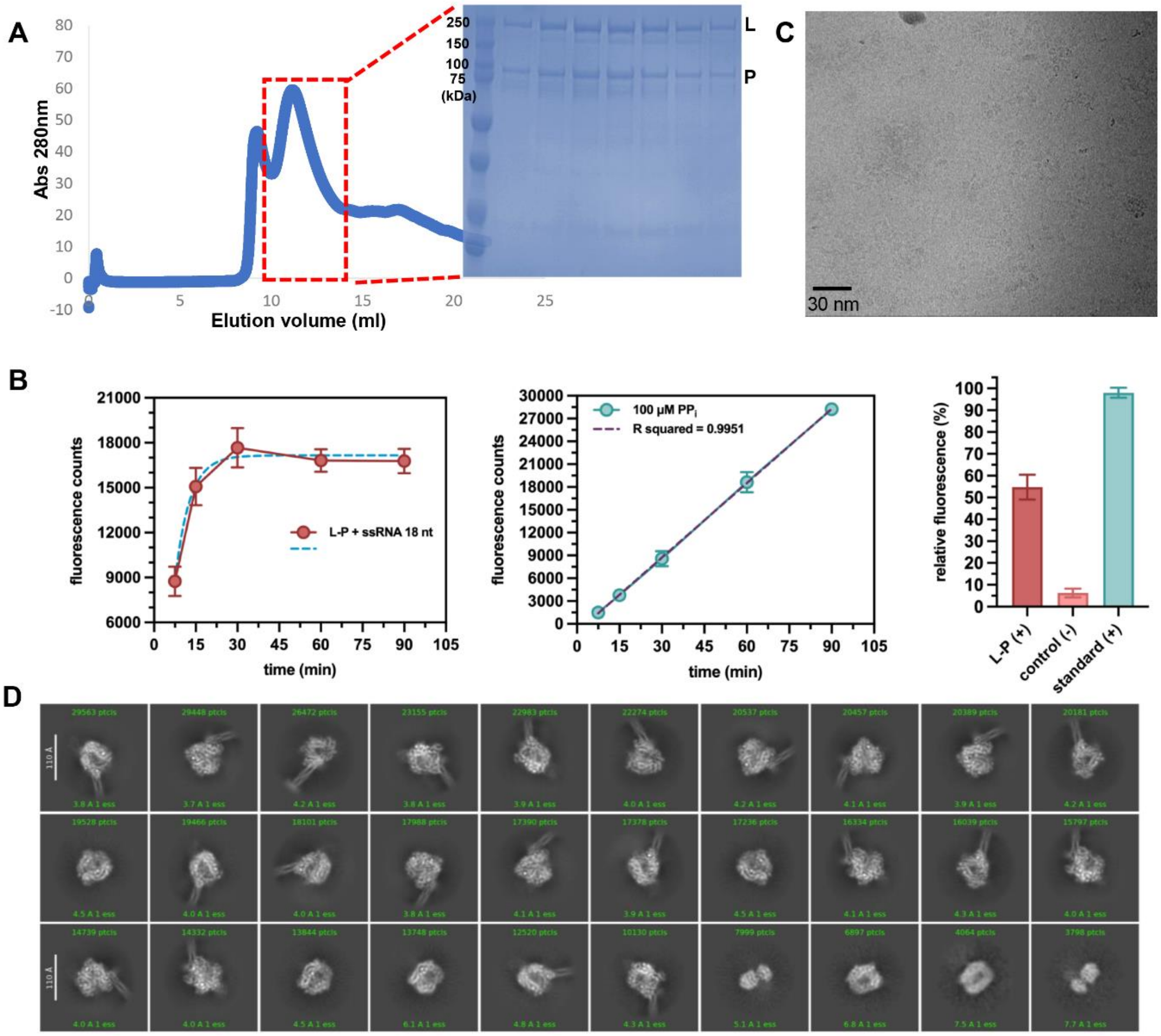

Fig. S1. Purification and cryo-EM analysis of the Nipah virus L-P complex. (**A**) Gel-filtration and SDS-PAGE analyses of the recombinant Nipah virus L-P complex. The fractions were loaded onto a 10% resolving gel and stained with Coomassie blue. The L and P bands are labeled on the right. (**B**) Time course of *in vitro* pyrophosphate (PP_i) assay (left panel) showing *de novo* RNA synthesis initiation of 18-nt ssRNA (red circles); the specific RdRp activity of Nipah virus L-P complex with respect to a PP_i hydrolysis standard reaction (middle panel, green circles) and a negative control reaction in the absence of L-P (right panel) is shown as percentage of relative fluorescence for the 90 min time point (right panel). Experimental data are the mean and standard deviations of at least four independent replicates. (**C**) A representative electron micrograph of the Nipah virus L-P complex. Scale bar, 300 Å. (**D**) Representative 2D class averages of the Nipah virus L-P particles.

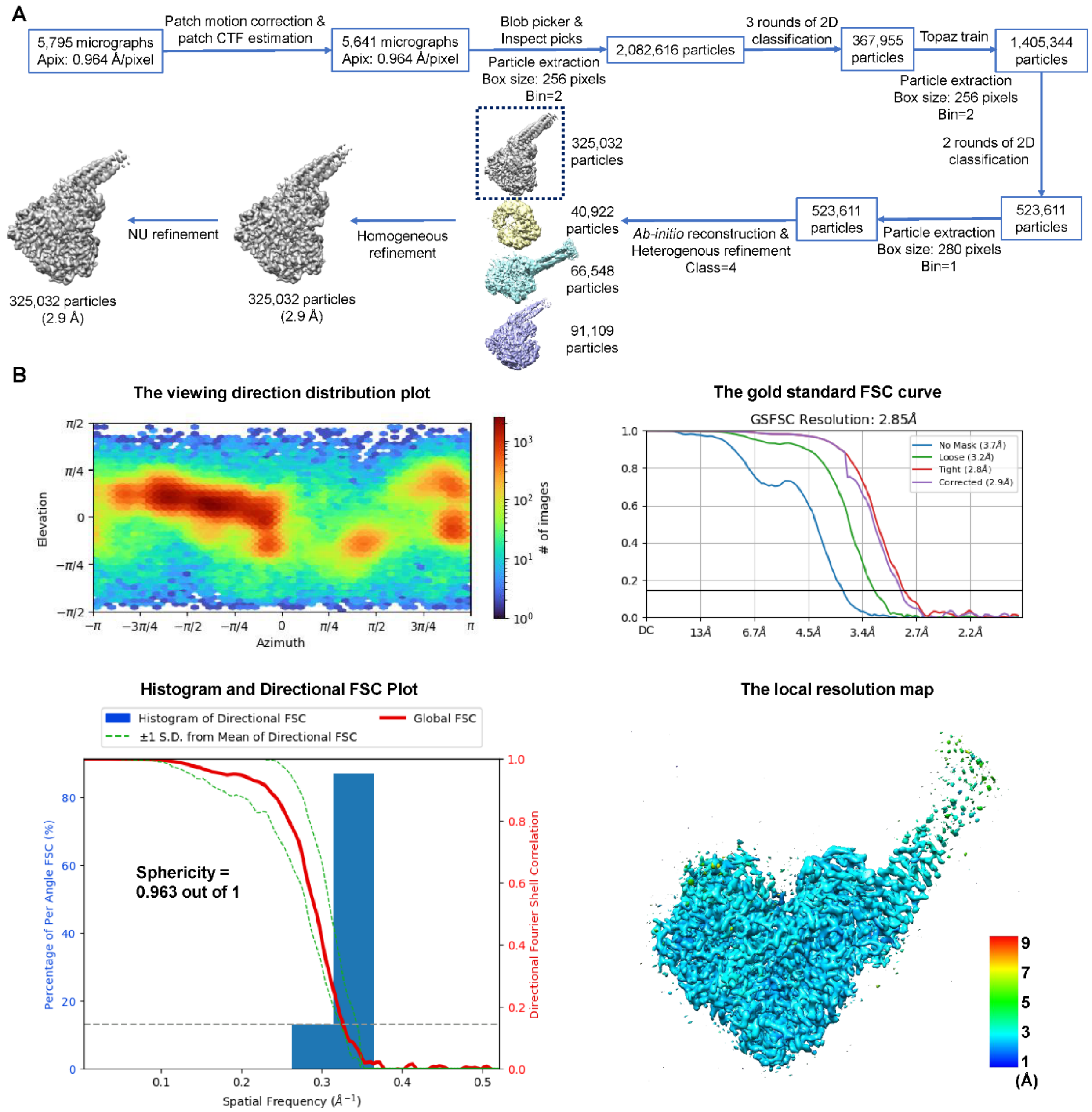

Fig. S2. Cryo-EM analysis of the Nipah virus L-P complex. (**A**) Flowchart of the cryo-EM data processing for the Nipah virus L-P complex. (**B**) Assessment of the cryo-EM map's quality, including the viewing direction distribution plot, the 0.143 gold standard FSC curve generated using cryoSPARC, the 3DFSC plot generated using 3DFSC (https://3dfsc.salk.edu/), and the color-coded local resolution map.

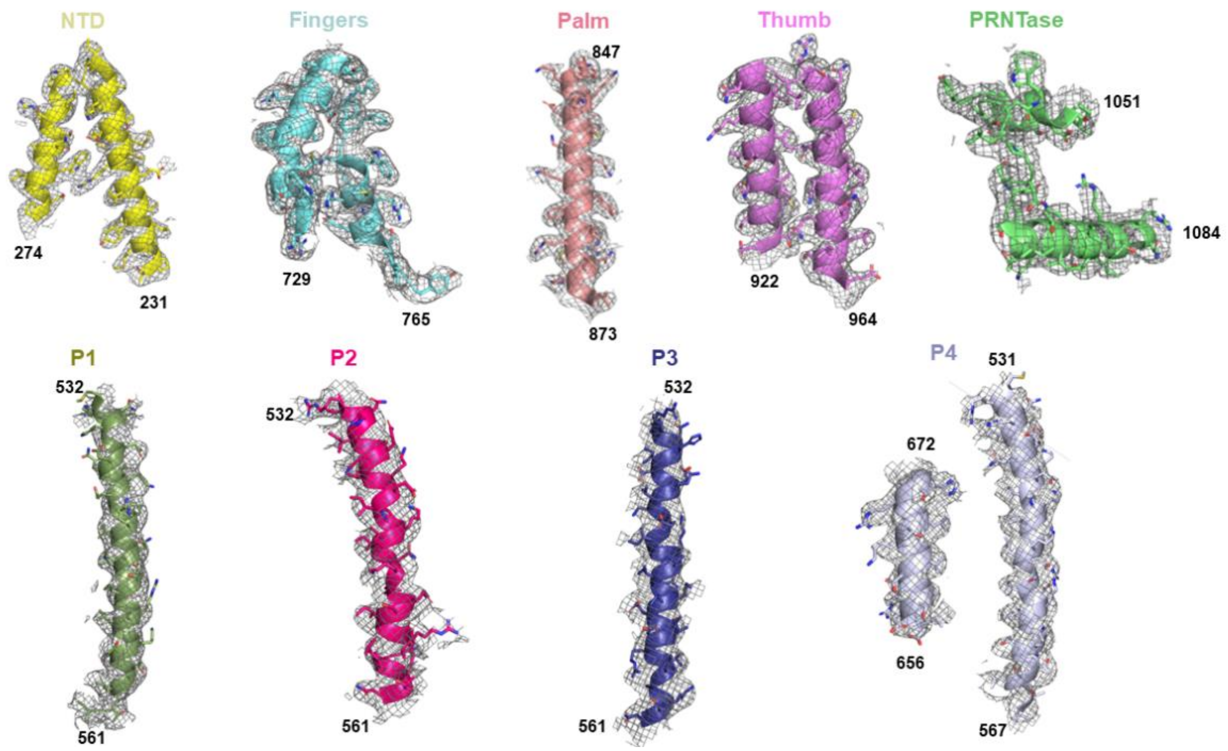

Fig. S3. Examples of cryo-EM density of the Nipah-L and P proteins. Models of Nipah-L and tetrameric P proteins were fitted into the density map using PyMOL, with the EM density for representative regions of each subunit displayed in mesh.

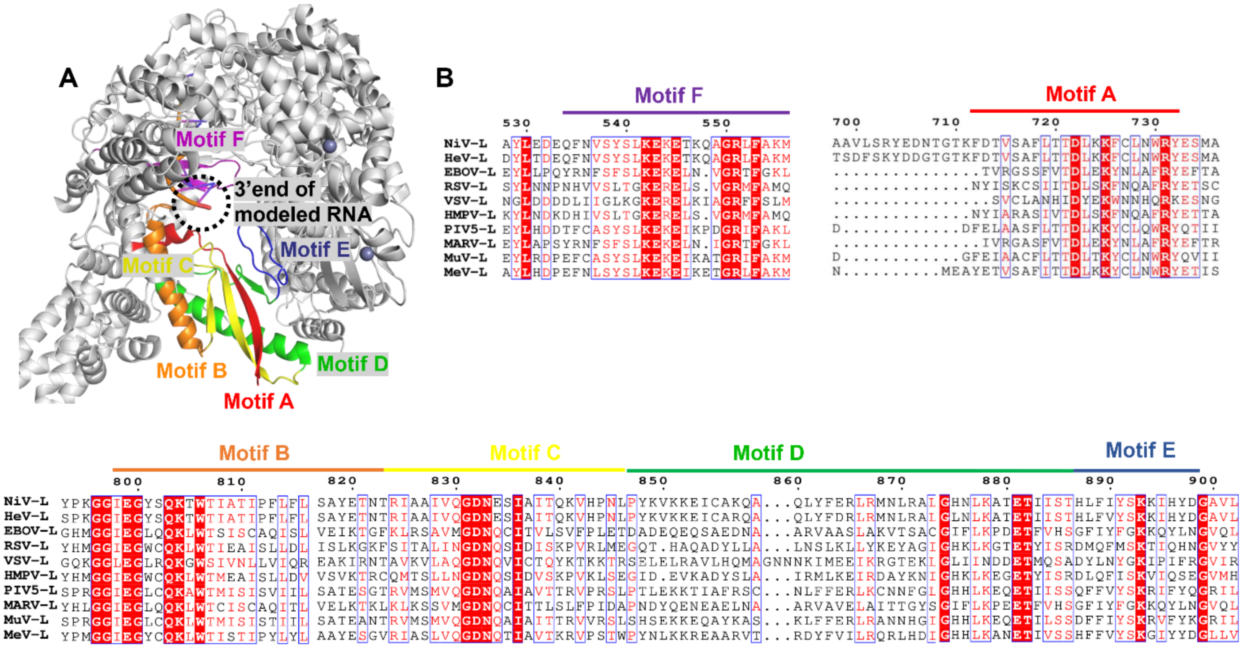

Fig. S4. Conserved amino acids in motifs A-F. (**A**) Structural details of the motifs A-F and the modeled RNA. Motifs A-F are color-coded, and the 3' end of modeled RNA is indicated with a black dashed circle and labeled. (**B**) Sequence alignment of the motifs A-F in Nipah virus L protein with those of other representative nsNSVs. Motifs A-F are labeled on the top of sequences.

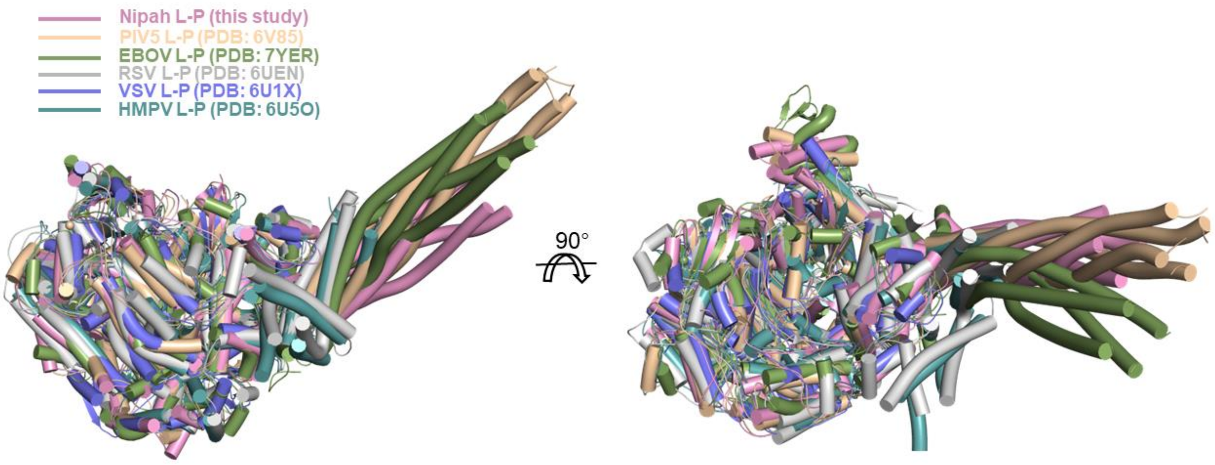

Fig. S5. Structural comparison of the Nipah virus L-P complex with its counterparts in PIV5, EBOV, RSV, VSV, and HMPV. The structures of different viruses' L-P complexes are color-coded and shown in cartoon representation with cylindrical helices and smooth loops.

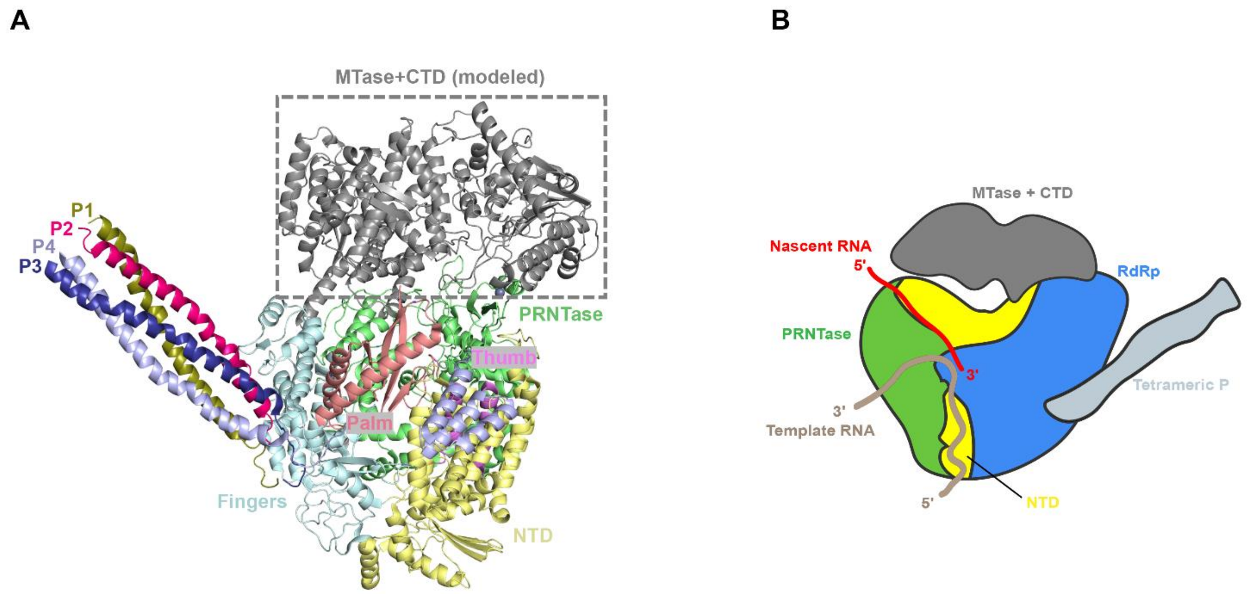

Fig. S6. Predicted model for the full-length Nipah virus L-P complex. (**A**) The invisible MTase and CTD domains of Nipah-L are indicated with a black dashed box. (**B**) Predicted RNA paths within the full-length Nipah L-P complex. The different domains of the L protein, the tetrameric P proteins, the template RNA, and the nascent RNA are color-coded.

Table S1. Cryo-EM data collection, refinement, and validation statistics.

Complex	Nipah virus L-P
EMDB	EMD-60355
PDB	8ZPV
**Data collection and processing**	
Magnification	130,000
Voltage (kV)	300
Electron exposure ($e^-/Å^2$)	50
Defocus range (-µm)	1.3-2.3
Pixel size (Å)	0.9643 (counting mode)
Symmetry imposed	C1
Initial particle images (no.)	2,082,616
Final particle images (no.)	325,032
Map resolution (Å)	2.9
FSC threshold	0.143
Map resolution range (Å)	2.4-3.5
**Refinement**	
Initial model used	AlphaFold2-predicted
Model resolution (Å)	2.9
FSC threshold	0.143
Model resolution range (Å)	2.4-3.5
Map sharpening B factor ($Å^2$)	132.6
Model composition	
Non-hydrogen atoms	12,440
Protein residues	1,552
Ligands	2
B factor ($Å^2$)	
Protein	162.1
Ligand	200.1
R.m.s. deviations	
Bond lengths (Å)	0.005
Bond angles (°)	0.694
Validation	
MolProbity score	2.90
Clashscore	13.1
Poor rotamers (%)	0.2
Ramachandran plot	
Favored (%)	91.4
Allowed (%)	8.5
Disallowed (%)	0.1
